# Supplementary material for: Novel Scintillating Nanoparticles for Potential Application in Photodynamic Cancer Therapy
Source: Pharmaceutics. 2022 Oct 22;14(11):2258. doi: 10.3390/pharmaceutics14112258 (PMC9697386; doi:10.3390/pharmaceutics14112258)
Supplement: Supplementary file 1 [file pharmaceutics-14-02258-s001.zip › pharmaceutics-1889010-supplementary.pdf]

Novel Scintillating Nanoparticles for Potential Application in Photodynamic Cancer Therapy

Bianca A. da Silva [1], Michael Nazarkovsky [1], Helmut Isaac Padilla-Chavarría [1], Edith Alejandra C. Mendivelso [2], Heber L. de Mello [2,3], Cauê de S. C. Nogueira [4], Rafael dos S. Carvalho [5], Marco Cremona [5], Volodymyr Zaitsev [1], Yutao Xing [4], Rodrigo da C. Bisaggio [2,3], Luiz A. Alves [2,*] and Jiang Kai [1]

[1]Chemistry Department, Pontifical Catholic University of Rio de Janeiro, 225 Marquês de São Vicente Str., Rio de Janeiro 22451-900, RJ, Brazil.

[2]Laboratory of Cellular Communication, Oswaldo Cruz Institute, Fiocruz, 4365 Brasil Av., Manguinhos, Rio de Janeiro 21040-360, RJ, Brazil.

[3]Biotechnology Department, Federal Institute of Rio de Janeiro, 121 Senador Furtado Str., Maracanã, Rio de Janeiro 20270-021, RJ, Brazil.

[4]High-resolution Electron Microscopy Lab, Advanced Characterization Center for Petroleum Industry (LaMAR/CAIPE), Fluminense Federal University, Niteroi 24210-346, RJ, Brazil.

[5]Physics Department, Pontifical Catholic University of Rio de Janeiro, 225 Marquês de São Vicente Str., Rio de Janeiro 22451-900, RJ, Brazil.

*Correspondence: alveslaa@gmail.com

Scanning Electron Microscopy

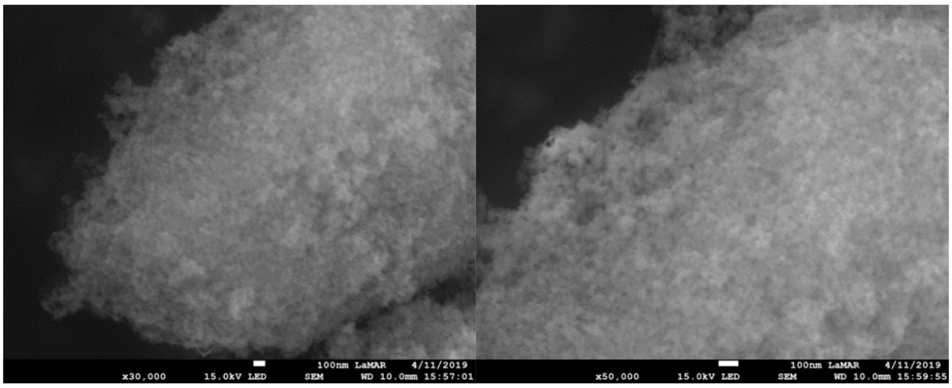

(a)

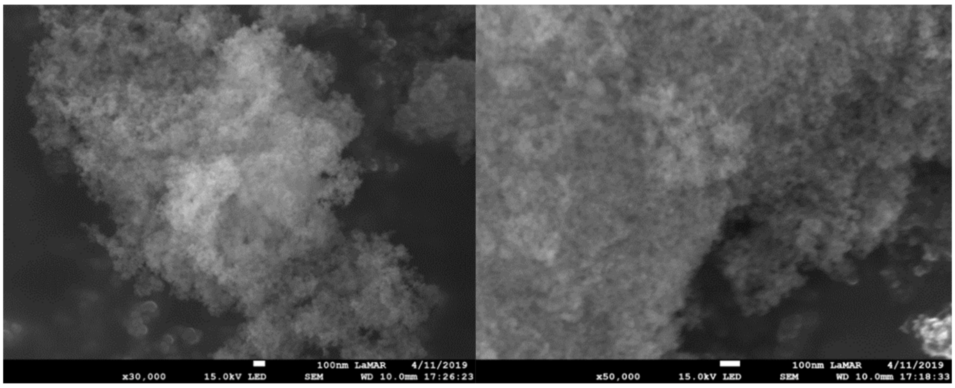

(b)

Figure S1: SEM images of (a) SiO_2-Gd_2O_3:Eu^{3+}(3%) and (b) SiO_2-Gd_2O_3:Eu^{3+}(5%).

Transmission Electron Microscopy

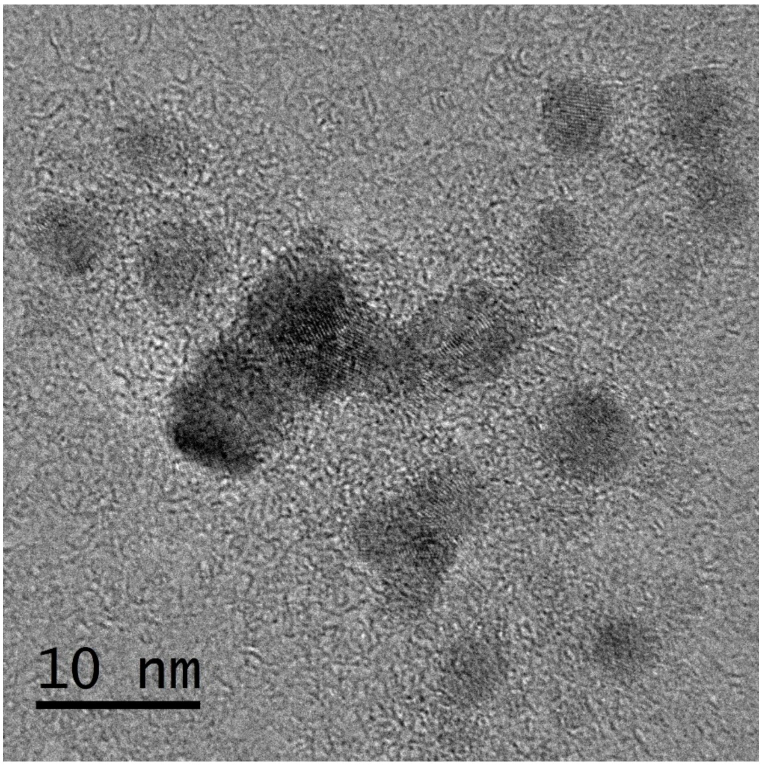

Figure S2: TEM image of SiO_2-Gd_2O_3:Eu^{3+}(1%), showing the crystallinity of the rare earth oxides.

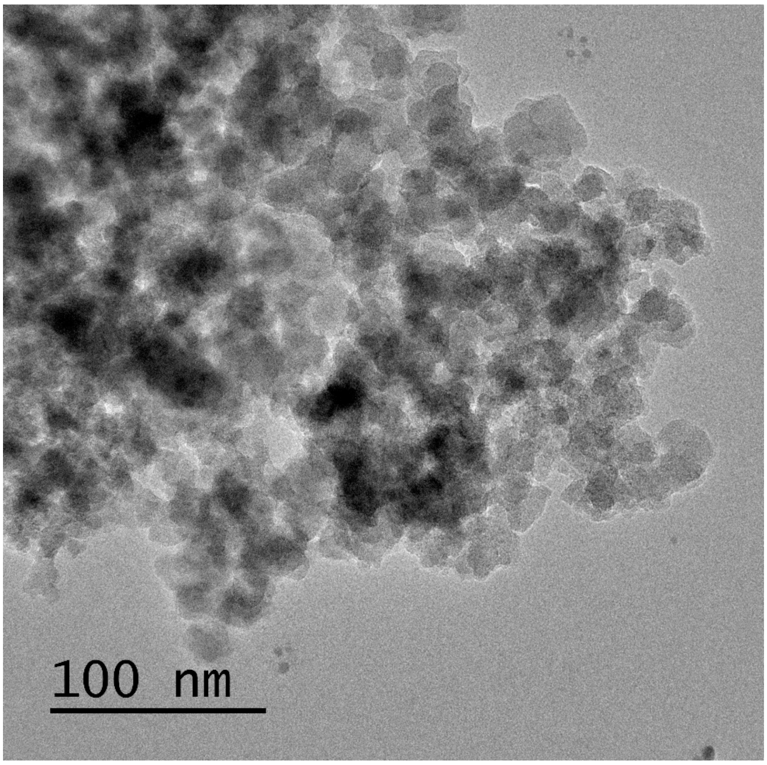

Figure S3: Low magnification TEM image showing the overall morphology of SiO_2-Gd_2O_3:Eu^{3+}(1%).

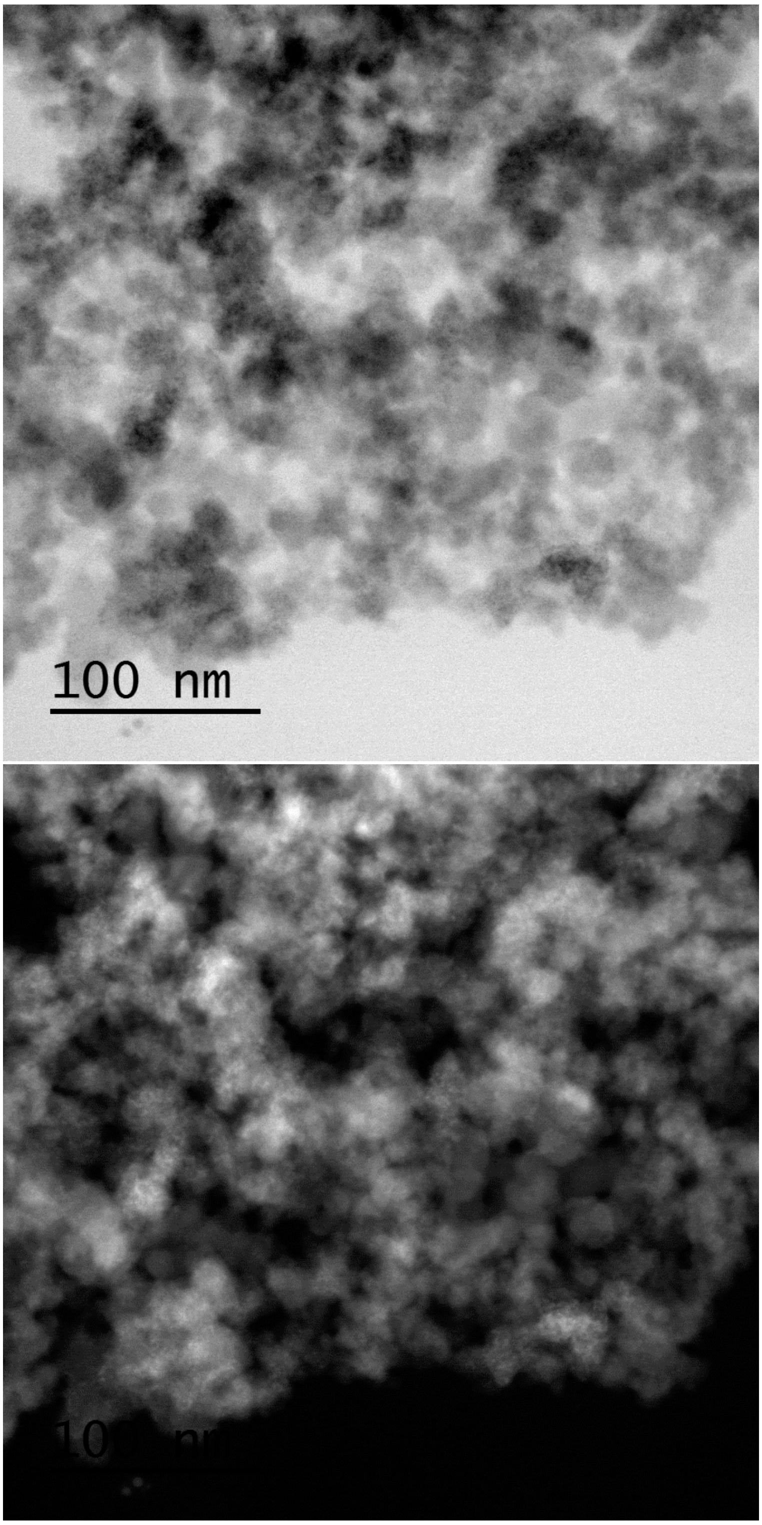

Figure S4: Upper: bright-field and lower: HAADF STEM images of SiO_2-Gd_2O_3:Eu^{3+}(1%). The atomic number contrast shows that the RE metal oxides are on the surface of the silica nanoparticles.

Luminescence Property

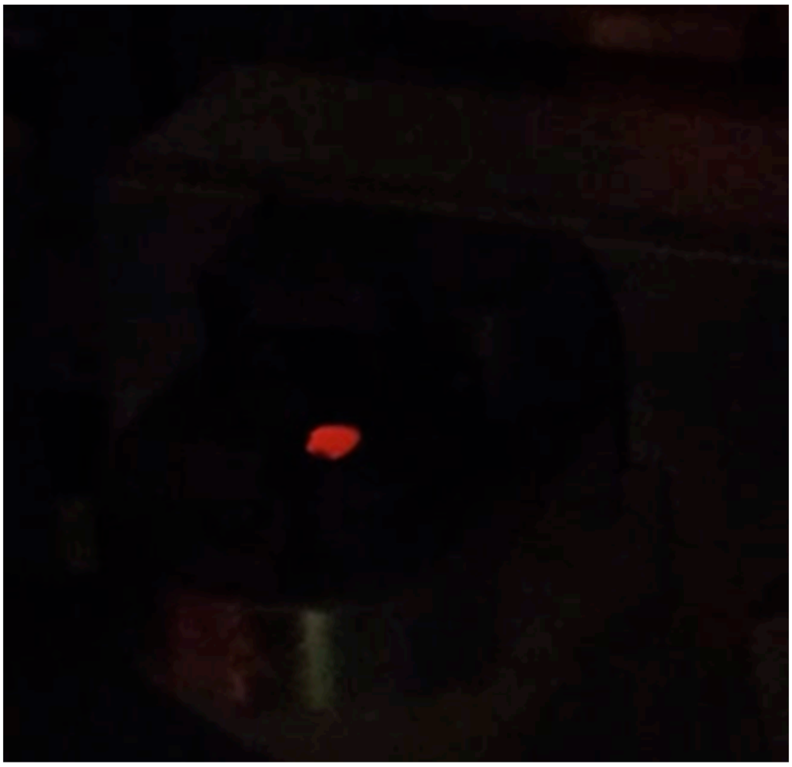

Figure S5: Scintillating effect of the $Gd_2O_3:Eu^{3+}$ nanoparticles emitting red light under X-ray irradiation.

Dynamic Light Scattering

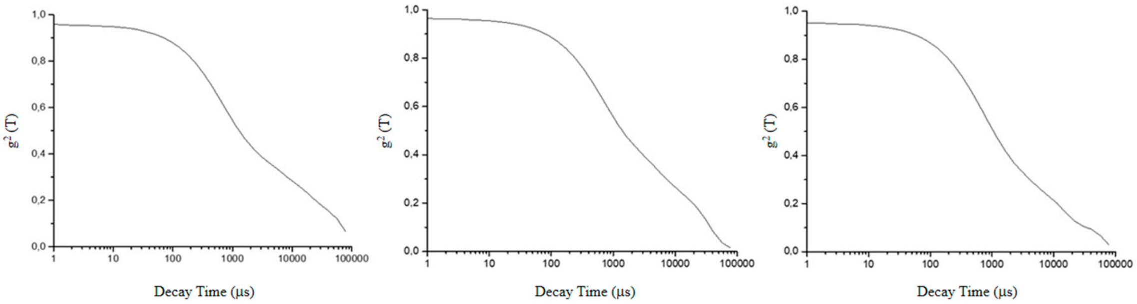

Figure S6: Autocorrelation functions referring to DLS measurements of $Gd_2O_3:Eu^{3+}$ nanoparticles.

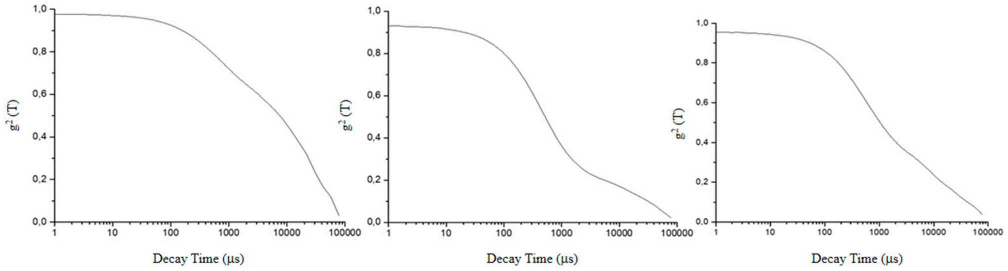

Figure S7: Autocorrelation functions referring to DLS measurements of SiO_2-$Gd_2O_3:Eu^{3+}$ nanoparticles.
